# Dysregulated hyaluronan metabolism drives inflammation and angiogenesis in proliferative diabetic retinopathy

**DOI:** 10.3389/fimmu.2026.1724199

**Published:** 2026-02-18

**Authors:** Ahmed M. Abu El-Asrar, Mohd I. Nawaz, Ajmal Ahmad, Mairaj Siddiquei, Eef Allegaert, Priscilla W. Gikandi, Gert De Hertogh, Ghislain Opdenakker

**Affiliations:** 1 Department of Ophthalmology, College of Medicine, King Saud University, Riyadh, Saudi Arabia; 2 Arab National Bank Research Chair in Ophthalmology, College of Medicine, King Saud University, Riyadh, Saudi Arabia; 3 Laboratory of Histochemistry and Cytochemistry, University of Leuven, Leuven, Belgium; 4 University Hospitals UZ Gasthuisberg, Leuven, Belgium; 5 Laboratory of Immunobiology, Department of Microbiology, Immunology and Transplantation, Rega Institute, University of Leuven, Leuven, Belgium; 6 Royal Academy of Medicine of Belgium (KAGB), Brussels, Belgium

**Keywords:** angiogenesis, endothelial cells, hyaluronan, inflammation, Müller cells, proliferative diabetic retinopathy

## Abstract

**Purpose:**

To investigate the expression levels of enzymes and receptors of the hyaluronan (HA) pathway, including HA synthase (HAS)-2, hyaluronidase (Hyal)-1, Hyal-2, CD44 and receptor for HA-mediated motility (RHAMM) in the ocular microenvironment of patients with proliferative diabetic retinopathy (PDR) and the role of HA pathway in inflammation and angiogenesis that drive PDR initiation and progression.

**Methods:**

Epiretinal fibrovascular membranes from PDR patients, vitreous samples from PDR and nondiabetic patients, rat retinas, retinal Müller glial cells and human retinal microvascular endothelial cells (HRMECs) were studied by immunohistochemistry, ELISA, Western blot analysis and spectrofluorometric analysis. Functional studies included evaluation of *in vivo* blood-retinal barrier integrity and analysis of *in vitro* cell adhesion and angiogenesis.

**Results:**

HA, HAS2, Hyal-1, Hyal-2, CD44, syndecan-1 and heparan sulphate levels were upregulated in PDR vitreous samples. Immunohistochemical analysis revealed expression of HAS2, Hyal-2, CD44 and RHAMM in epiretinal membranes, with significant positive correlations between angiogenic activity and HAS2, Hyal-2 and CD44 expression. Diabetes upregulated Hyal-1, CD44, RHAMM and reactive oxygen species in rat retinas. Intravitreal administration of ultralow molecular weight HA (ULMW-HA) in normal adult rats increased retinal vascular permeability and induced upregulation of phospho-NF-κB, phospho-ERK1/2, VEGF, HMGB1, ICAM-1 and VCAM-1 protein levels. In Müller cell cultures, ULMW-HA induced upregulation of phospho-ERK1/2, phospho-NF-κB, HMGB1, VEGF, angiopoietin-2 and MCP-1/CCL2 proteins. The ERK1/2 inhibitor U-0126 and the NF-κB inhibitor BAY11–7085 attenuated ULMW-HA–induced upregulation of VEGF, angiopoietin-2 and MCP-1/CCL2 levels. The hyaluronidase inhibitor apigenin reduced the levels of VEGF and MCP-1/CCL2 induced by diabetic mimetic conditions. In cultured HRMECs, ULMW-HA induced cell migration, whereas apigenin attenuated shedding of soluble syndecan-1 induced by diabetic mimetic conditions and reduced TNF-α–induced upregulation of ICAM-1, VCAM-1 and adherence of monocytes.

**Conclusions:**

Abnormal HA metabolism is involved in diabetes-induced retinal endothelial dysfunction and ULMW-HA drives inflammation and angiogenesis in PDR.

## 1 Introduction

Diabetic retinopathy is a serious and most frequent microvascular complication of diabetes mellitus in comparison with neuropathy and nephropathy. Abundant evidence exists for key roles of oxidative stress and inflammation in the pathogenesis of diabetic retinopathy (1–3). Enhanced adhesion of circulating leukocytes to the retinal microvascular endothelium is observed as an early change associated with diabetic retinopathy and actively contributes to chronic low-grade subclinical inflammatory vasculopathy and capillary nonperfusion. Retinal leukocyte stasis and the adhesion of leukocytes to the endothelium correlate with increased expression of retinal intercellular adhesion molecule-1 (ICAM-1) and the leukocyte integrin CD18 (4). Oxidative stress and inflammation contribute to the pathogenesis of diabetes-induced retinal endothelial cell dysfunction and breakdown of the blood-retinal barrier leading to increased retinal microvascular permeability (1–4). In addition, ischemia-induced retinal angiogenesis leading to pathologic growth of new blood vessels, infiltration of leukocytes and proliferation of myofibroblasts are critical for the initiation and progression of proliferative diabetic retinopathy (PDR). Several studies demonstrated overexpression of proinflammatory, proangiogenic and fibrogenic factors in the ocular microenvironment of patients with PDR (5–7). These findings suggest that inflammation, angiogenesis and fibrosis are hallmarks of PDR initiation and progression.

The glycosaminoglycan hyaluronic acid (HA), or hyaluronan, is a major component of the extracellular matrix (ECM). HA is synthesized as a large negatively charged polymer of disaccharide units, consisting of D-glucuronic acid and N-acetyl-D-glucosamine, one unit corresponding to about 400 Daltons (Da). HA is also the major glycosaminoglycan in the human vitreous body and is a prominent constituent of the interphotoreceptor matrix in the retina (8, 9). HA biosynthesis takes place at the plasma membrane and is catalyzed by three enzymes named HA synthases (HAS) 1, 2, and 3. HAS2 is the main enzyme responsible for excessive HA production. Overexpression of HAS2 increases tumor HA production and enhances tumor growth and angiogenesis (10, 11). HA is synthesized as a high-molecular weight (HMW-HA) polymer with a molecular mass ranging from 1000 to 6000 kilodaltons (kDa), corresponding to approximately 2,500 to 15,000 disaccharide units. When ECM homeostasis is disrupted by pathological conditions, such as inflammation, fibrosis and cancer, endogenous HMW-HA is fragmented faster than under homeostatic conditions by hyaluronidases and reactive oxygen species (ROS). This altered turnover in cancer, fibrosis and inflammation unbalances the equilibrium towards a higher concentration of low-molecular weight (LMW-HA) species, with a molecular mass ≤ 250 kDa, corresponding to less than 625 disaccharide units (12, 13). It has been suggested that hyaluronidase (Hyal)-1 and Hyal-2 are the major hyaluronidases and that they act in concert to degrade HMW-HA to LMW-HA (12–14). Importantly, the biological activities of HA change depending on its molecular weight. Under healthy conditions, HMW-HA predominates, exhibiting anti-inflammatory and anti-angiogenic functions, whereas LMW-HA fragments predominate under pathological conditions and are highly pro-inflammatory and pro-angiogenic (12–14). The biological effects of LMW-HA are mediated through the cell surface receptors cluster of differentiation (CD)44 and receptor for hyaluronan-mediated motility (RHAMM), which is designated as CD168, activating cellular signals for inflammation and angiogenesis (12, 15).

In addition, HA is a major component of endothelial glycocalyx (EG), which is the layer that covers the luminal surface of blood vessels on the apical surface of endothelial cells. The EG is mainly composed of glycosaminoglycans and proteoglycans (14, 16). The glycocalyx acts as a regulator of vascular permeability, modulates leukocyte and platelet adhesion to endothelial cells and inhibits coagulation. HA is essential to maintain the integrity of the EG functional barrier. The EG is damaged in several inflammatory and ischemic conditions, leading to dysfunction of endothelial cells and resulting in increased vascular permeability and increased leukocyte and platelet adhesion inducing a pro-thrombotic state. EG damage and degradation are accompanied by shedding of the EG components syndecan-1, heparan sulphate and HA, which are used as biomarkers of EG degradation (14, 16).

Until now the HA pathway has been mainly studied in cancer and little is known about the role of the HA pathway in PDR initiation and progression. Understanding the mechanisms involved in abnormal HA metabolism and signaling in PDR could have important implications for the design of novel therapeutic approaches. Therefore, we investigated the expression of members of the HA pathway, including HAS2, Hyal-1, Hyal-2, CD44 and RHAMM in the ocular microenvironment of patients with PDR and the functional role of HA fragmentation in inflammation and angiogenesis that drive PDR initiation and progression.

## 2 Materials and methods

### 2.1 Vitreous samples and epiretinal membranes specimens

Undiluted vitreous fluid samples (0.3–0.6 ml) were obtained from 40 patients with PDR during pars plana vitrectomy, for the treatment of tractional retinal detachment, and/or nonclearing vitreous hemorrhage and processed as described previously (5–7). The control vitreous samples were from a group of 32 patients who had undergone vitrectomy for the treatment of rhegmatogenous retinal detachment with no proliferative vitreoretinopathy. These 32 patients were clinically checked to be free from diabetes or other systemic disease and were compared with PDR patients. Epiretinal fibrovascular membranes were obtained from 14 patients with PDR during pars plana vitrectomy for the repair of tractional retinal detachment. The epiretinal membranes were processed as previously described (5–7).

### 2.2 Immunohistochemical staining for epiretinal membranes

The immunohistochemical and the sequential double immunohistochemical stainings were performed using the Leica Bond Max autostainer system (M496834, Leica, Diegem, Belgium) with Bond Polymer Refine Red Detection kit (DS9390, Leica) and Bond Polymer Refine Detection kit (DS9800, Leica) as recently detailed (17).

For CD31, α-smooth muscle actin (α-SMA), HAS2 and CD44 detection, antigen retrieval was performed by boiling (99°C) the sections in citrate based buffer [pH 5.9–6.1] [ER1-AR9961, Bond Epitope Retrieval Solution 1; Leica] for 20 minutes. For CD45, CD68, Hyal-2 and RHAMM (CD168) detection, antigen retrieval was performed by boiling (99°C) the sections in Tris/EDTA buffer [pH 9] [ER2-AR9640, Bond Epitope Retrieval Solution 2; Leica] for 20 minutes. Subsequently, the sections were incubated for 60 minutes with mouse monoclonal anti-CD31 (ready-to-use; clone JC70A; Dako, Glostrup, Denmark), mouse monoclonal anti-CD45 (ready-to-use; clones 2B11+PD7/26; Dako), mouse monoclonal anti-CD68 antibody (ready-to-use; clone KP1; Dako), mouse monoclonal antibody against α-SMA (ready-to-use; clone 1A4; Dako), mouse monoclonal antibody against HAS2 (1:1000; ab140671; Abcam, Cambridge, UK), rabbit polyclonal antibody against Hyal-2 (1:250; ab68608; Abcam), mouse monoclonal anti-CD44 antibody (1:150; Cat No 3570; Cell Signaling Technology, Danvers, MA, USA) and rabbit polyclonal antibody against RHAMM (CD168) (1:250; ab124729; Abcam).

To identify the phenotype of cells expressing HAS2, CD44 and RHAMM (CD168), sequential double immunohistochemistry was performed as described (17). The sections were incubated for 60 mins with the first primary antibodies (anti-CD45 or CD68) and subsequently treated with peroxidase-conjugated secondary antibody to define the leukocytes. The resulting immune complexes were visualized by enzymatic reaction of the 3, 3’-diaminobenzidine tetrahydrochloride substrate. Incubation of the second primary antibodies (anti-HAS2, CD44 and RHAMM) for 60 mins was followed by treatment with alkaline phosphatase-conjugated secondary antibody for 30 mins and fast red reactions for 2 times 15 mins. No counterstain was applied. Negative controls were by omission of the primary antibody from the staining protocol. Instead, the ready-to-use DAKO Real antibody Diluent (Agilent Technologies Product Code 52022) was applied.

### 2.3 Quantitation

The level of vascularization in epiretinal fibrovascular membranes was determined by immunodetection of the vascular endothelium marker CD31. Immunoreactive blood vessels and cells were counted in five representative fields, with the use of an eyepiece with a calibrated grid in combination with the 40x objective lens. These representative fields were selected based on the presence of immunoreactive blood vessels and cells. With this magnification and calibration, immunoreactive blood vessels and cells present in an area of 0.33 mm × 0.22 mm were counted. We did not use automated image analysis systems. The vessel and cell countings were done by the lead author and the immunohistochemical analysis was done in a disease-blinded way by an expert histopathologist.

### 2.4 Induction of streptozotocin-induced diabetes in rats

All procedures with animals were performed in accordance with the Association for Research in Vision and Ophthalmology (ARVO) statement for use of animals in ophthalmic and vision research and were approved by the institutional animal care and use committee of the College of Pharmacy, King Saud University. As described previously (5–7), adult male Wistar rats of 10–12 weeks of age (200–220 g) were fasted overnight, and a single-bolus dose of streptozotocin of 60 mg/kg in 10 mM sodium citrate buffer, pH 4.5, (Sigma, St. Louis, MO, USA) was injected intraperitoneally. Equal volumes of citrate buffer were injected in age-matched control rats. Rats were considered diabetic if their blood glucose levels were in excess of 250 mg/dL. After 4-weeks of diabetes, the rats were euthanized using 100% carbon dioxide, the eyes were removed, and retinas were isolated and frozen immediately in liquid nitrogen and stored at - 80°C until analyzed. Similarly, retinas were obtained from age-matched nondiabetic control rats.

### 2.5 Intravitreal injection of ULMW-HA

Male Wistar rats (200–220 g) were kept under deep anesthesia using intraperitoneal injection of 350 mg/kg chloral hydrate, and a sterilized solution of commercially available and biochemically defined Hyaluronan (Ultra Low MW hyaluronic acid/ULMW-HA) (5 ng, 25 ng or 50 ng in 5 μl; Cat No GLR003, R&D Systems, Minneapolis, MN, USA) was injected into the vitreous of the right eye as previously described (5, 6). This ULMW-HA ranges in molecular mass between 4 to 8 kDa and thus corresponds to about 10 to 20 disaccharide units. For the control, the left eye received 5 μl of sterile phosphate buffer saline (PBS). The animals were sacrificed (with the use of 100% carbon dioxide) 5 days after intravitreal administration, and the retinas were carefully dissected, snap frozen in liquid nitrogen, and stored at -80°C until analyzed.

### 2.6 Measurement of blood-retinal barrier breakdown

Blood-retinal barrier (BRB) breakdown in excised retinas was evaluated 5 days after intravitreal injection as previously described (5, 6). BRB breakdown was calculated using the following equation, with the results being expressed in µl/(g∗h).


$$\frac{Retinal\;FITC-dextran\;(\text{µ}g)/retinal\;weight\;(g)}{Plasma\;FITC-dextran\;concentration(µg/ul)*circulation\;time\;(h)}$$

### 2.7 Human retinal Müller glial cell and human retinal microvascular endothelial cell cultures

The human retinal Müller glial cell line (MIO-M1) (a generous gift from Prof. A. Limb, Institute of Ophthalmology, University College London, UK) and commercially available human retinal microvascular endothelial cells (HRMECs) (Cell Systems Corporation, Kirkland, WA, USA) were cultured as described previously (5–7).

The following stimuli were used to mimic diabetic conditions: treatment of Müller cells or HRMECs with 5 ng/ml recombinant human tumor necrosis factor-alpha (TNF-α) (Cat No 210-TA, R&D Systems), 300 μM of the hypoxia mimetic agent cobalt chloride (CoCl_2_) (Cat No A1425-L, Avonchem Limited, UK), or 10 mM H_2_O_2_, or 25mM glucose (Cat No GL0125100, Scharlau S.L, Gato Prez, Spain) for 16 h. To compensate for osmotic effects when using 25mM glucose as high-glucose (HG) treatment, 25 mM mannitol (Cat No MA01490500, Scharlau S.L, Gato Prez, Spain) was used as a control.

Additionally, overnight starved Müller cells were treated with hyaluronan (ULMW-HA at 50 μg/ml, Cat No GLR003, R&D Systems) in the absence or presence of 1h pretreatment with the nuclear factor-kappa B (NF-κB) inhibitor BAY11-7085 (5 µM, Cat No sc-202490, Santa Cruz Biotechnology Inc., Santa Cruz, CA USA), or the ERK1/2 inhibitor U0126 (5 µM, Cat No sc-222395A, Santa Cruz Biotechnology Inc.) or the combination of the NF-κB inhibitor BAY11–7085 and the ERK1/2 inhibitor U0126 for 16 hours. Müller cells or HRMECs were pretreated for 1h with apigenin (Cat No 1227/10, R&D Systems) followed by treatment with 5 ng/ml TNF-α, 300 μM CoCl_2_, or 25mM glucose for 16 h.

After the treatments as detailed above, cell supernatants were collected and processed for ELISA analysis. Harvested cells were lysed in a radioimmunoprecipitation assay (RIPA) lysis buffer (sc-24948, Santa Cruz Biotechnology, Inc.) for Western blot analysis.

### 2.8 Enzyme-linked immunosorbent assays

Enzyme-linked immunosorbent assay (ELISA) kits for human Hyal-1 (Cat No DY7358), human Hyaluronan (Cat No DY3614-05), and human syndecan-1 (Cat No DY2780), human vascular endothelial growth factor (VEGF) (Cat No DY293B), human monocyte chemoattractant protein-1 (MCP-1/CCL2) (Cat No DY279), human matrix metalloproteinase-9 (MMP-9) (Cat No DY911) and human angiopoietin-2 (Cat No DY623) were purchased from R&D Systems. An ELISA kit for human heparan sulphate (Cat No NBP3-31897) was purchased from Novus Biologicals, LLC (Centennial, USA).

Levels of Hyal-1, hyaluronan, syndecan-1 and heparan sulphate in vitreous fluid; and VEGF, MCP-1/CCL2, MMP-9, syndecan-1 and angiopoietin-2 in cell culture medium were determined using the aforementioned ELISA kits according to the manufacturer’s instructions. The minimum detection limits for heparan sulphate, Hyal-1, hyaluronan, syndecan-1, VEGF, MCP-1/CCL2, angiopoietin-2 and MMP-9 ELISA kits were approximately 50 pg/ml, 10 pg/ml, 150 pg/ml, 50 pg/ml, 12 pg/ml, 9 pg/ml, 10 pg/ml and 10 pg/ml, respectively.

### 2.9 Western blot analysis of human vitreous fluid, human retinal Müller glial cell and human retinal microvascular endothelial cell lysates and rat retinas

Retina and cell lysates were homogenized in Western blot lysis buffer [30 mM Tris-HCl; pH 7.5, 5 mM EDTA, 1% Triton X-100, 250 mM sucrose, 1 mM sodium vanadate, and a complete protease inhibitor cocktail from Roche (Mannheim, Germany)]. After centrifugation of the homogenates (14,000 X g for 15 min, 4°C), protein concentrations were measured in the supernatants (Bradford protein assay kit; Bio-Rad Laboratories, Hercules, CA, USA). Equal amounts (30–50 μg) of the protein extracts from lysates were subjected to SDS–PAGE and transferred onto nitrocellulose membranes.

To determine the presence of Hyal-1, Hyal-2, HAS2, CD44, syndecan-1, heparan sulphate and RHAMM in the vitreous samples, equal volumes (10 μL) of vitreous samples were boiled in Laemmli’s sample buffer (1:1, v/v) under reducing condition for 10 min.

Immunodetection was performed with the use of rabbit polyclonal anti-Hyal-1 antibody (1:1000, NBP2-16906, Novus Biologicals), mouse polyclonal anti-Hyal-2 antibody (1:1000, H00008692-B02P, Novus Biologicals), mouse monoclonal anti-HAS2 antibody (1:1000, ab140671, Abcam), rabbit monoclonal anti-CD44 antibody (1:1000, ab189524, Abcam), rabbit monoclonal anti-RHAMM antibody (1:1000, ab124729, Abcam), rabbit monoclonal anti-phospho-extracellular signal-regulated kinase (ERK)1/2 antibody (1:1000, MAB1018, R&D Systems), rabbit polyclonal anti-p65 subunit of nuclear factor-kappa B (phospho-NF-κB) (1:1000, NB100-82086, Novus Biologicals), rabbit polyclonal anti-high-mobility group box1 (HMGB1) (1:1000, Cat. no. ab18256, Abcam), mouse monoclonal anti-VEGF antibody (1:750, MAB293, R&D Systems), mouse monoclonal anti-intercellular adhesion molecule-1 (ICAM-1) antibody (1:100, sc-8439, Santa Cruz Biotechnology Inc.), and mouse monoclonal anti-vascular cell adhesion molecule-1 (VCAM-1) antibody (1:100, sc-13160, Santa Cruz Biotechnology Inc.).

Nonspecific binding sites on the nitrocellulose membranes were blocked (1.5 h, room temperature) with 5% non-fat milk made in Tris-buffered saline containing 0.1% Tween-20 (TBS-T). Three TBS-T washings (5 min each) were performed before the secondary antibody treatment at room temperature for 1 h. The secondary antibodies included goat anti-rabbit immunoglobulin (sc-2004) and goat anti-mouse immunoglobulin (1:2000, sc-2005, Santa Cruz Biotechnology Inc.). To verify equal loading, reacted antibodies were stripped and the membranes re-probed either with mouse monoclonal anti-β-actin-specific antibody (1:2000, sc-47778, Santa Cruz Biotechnology Inc.) or rabbit polyclonal anti-β-tubulin-specific antibody (1:2000, ab21058, Abcam). Bands were visualized with the use of high-performance chemiluminescence (G: Box Chemi-XX9 from Syngene, Synoptic Ltd., Cambridge, UK), and the band intensities were quantified with the use of GeneTools software (Syngene by Synoptic Ltd.).

### 2.10 Reactive oxygen species assay

Reactive oxygen species (ROS) generation was measured in retinal tissue homogenates using 2′-7′-dichlorofluorescein-diacetate (DCFH-DA) as described previously (17). Retinas were homogenized in PBS in the presence of protease inhibitor with the use of a glass homogenizer. Samples containing 20 μg protein diluted in PBS were incubated (15 minutes) in 5 μM DCFH-DA in the dark. Fluorescence was measured every 15 minutes for 1 hour with excitation and emission wavelengths of 488 nm and 525 nm, respectively.

### 2.11 Cell adhesion assay

To determine leukocyte adhesion onto stimulated HRMEC monolayers, we used the CytoSelect Leukocyte-endothelium adhesion kit (Cat. No. CBA-210, Cell Biolabs, Inc. San Diego, CA, USA). The assay protocol was followed as described previously (5, 7). Briefly, 2 x 10^5^ HRMECs (Cell Systems Corporation) were seeded into 0.2% gelatin-coated 24-well plates. After reaching a confluent monolayer, overnight starved endothelial cells were 1h pretreated with apigenin (Cat No 1227/10, R&D Systems) followed by 5 ng/ml TNF-α treatment for 16 h. Next, 5 x 10^5^ fluorescent-LeukoTracker labelled monocytic THP-1 cells (American Type Culture Collection, Manassas, VA, USA) were added on the top of treated HRMECs monolayer for 45 min. After washing 3 times, the remaining adherent THP-1 cells were lysed and fluorescence was measured using a spectraMax Gemini-XPS (Molecular Devices, CA, USA) with excitation and emission wavelengths of 485 nm and 538 nm, respectively.

### 2.12 *In vitro* migration assay

HRMECs were plated at 1 x 10^5^ cells/well on six-well culture plates and allowed to grow as described above till 80–90% confluency. Quiescence was induced by incubating the cells in minimal media overnight. Using sterile pipette tips, scratches were made, and then cells were rinsed with PBS. Cells were left untreated or treated with 10 ng/mL of recombinant VEGF or 100 µg/mL of Hyaluronan (ULMW-HA, Cat No GLR003, R&D Systems) for 16 h. Cell migration was monitored using an inverted microscope (Olympus IX81, Olympus Corporation, Tokyo, Japan). Analysis of migration was done using Image J software (http://imagej.nih.gov/ij/, version 1.53a).

### 2.13 Statistical analysis

Data collection and management were done in a database in Microsoft Excel 2010^®^ software. Data analysis and figures were prepared using SPSS^®^ version 21.0 (IBM Inc., Chicago, Illinois, USA). Normality tests were done using Q-Q plots and Shapiro-Wilk test and the data were normally distributed. Hence, the data were illustrated as bar charts showing means ± standard deviations (SD) or standard error of mean. To test the differences between the groups, One-Way ANOVA and Independent t-tests (applying Bonferroni correction where necessary) were done. Moreover, Pearson’s correlation analysis was carried out. Any output with a p-value below 0.05 was statistically significant.

## 3 Results

### 3.1 Expression of HAS2, Hyal-2, CD44 and RHAMM in epiretinal fibrovascular membranes from patients with PDR

**Figure 1:**
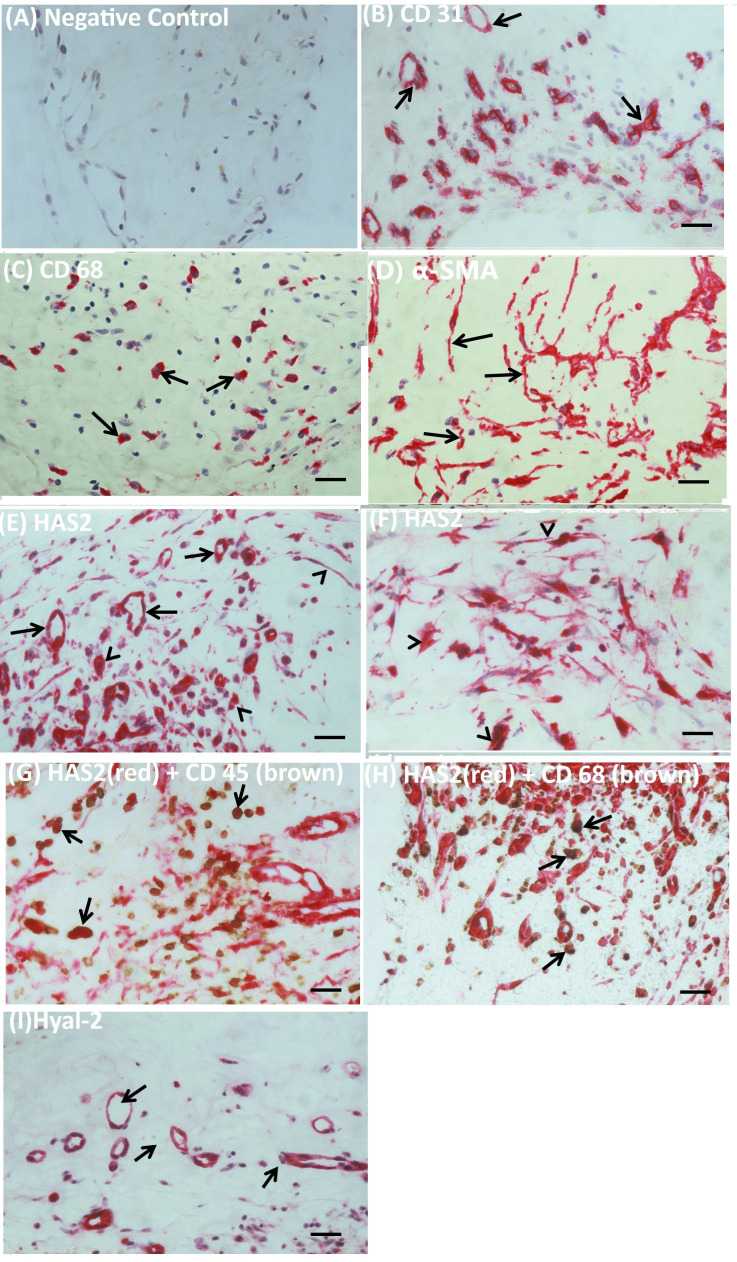
Immunohistochemical analysis of fibrovascular epiretinal membrane from patients with proliferative diabetic retinopathy. **(A)** Negative control slide (the incubation step with primary antibody was omitted) showing no staining. **(B)** Pathologic neovessels were detected by the endothelial cell marker CD31. **(C)** Monocytes/macrophages were detected by the CD68 marker. **(D)** Myofibroblasts were detected by the α-SMA marker. **(E, F)** Immunoreactivity for hyaluronan synthase (HAS)2 was detected in endothelial cells lining neovessels (arrows) and stromal cells (arrowheads). **(G)** Stromal cells were leukocytes co-expressing CD45 (arrows), **(H)**, monocytes/macrophages co-expressing CD68 (arrows) and **(F)** spindle-shaped cells (arrowheads). The spindle-shaped cells expressing HAS2 are most probably myofibroblasts expressing α-SMA [see **(D)**]. **(I)** Staining for hyaluronidase (Hyal)-2 showed immunoreactivity in endothelial cells lining neovessels (arrows) (black scale bar 10 µm).

**Figure 2:**
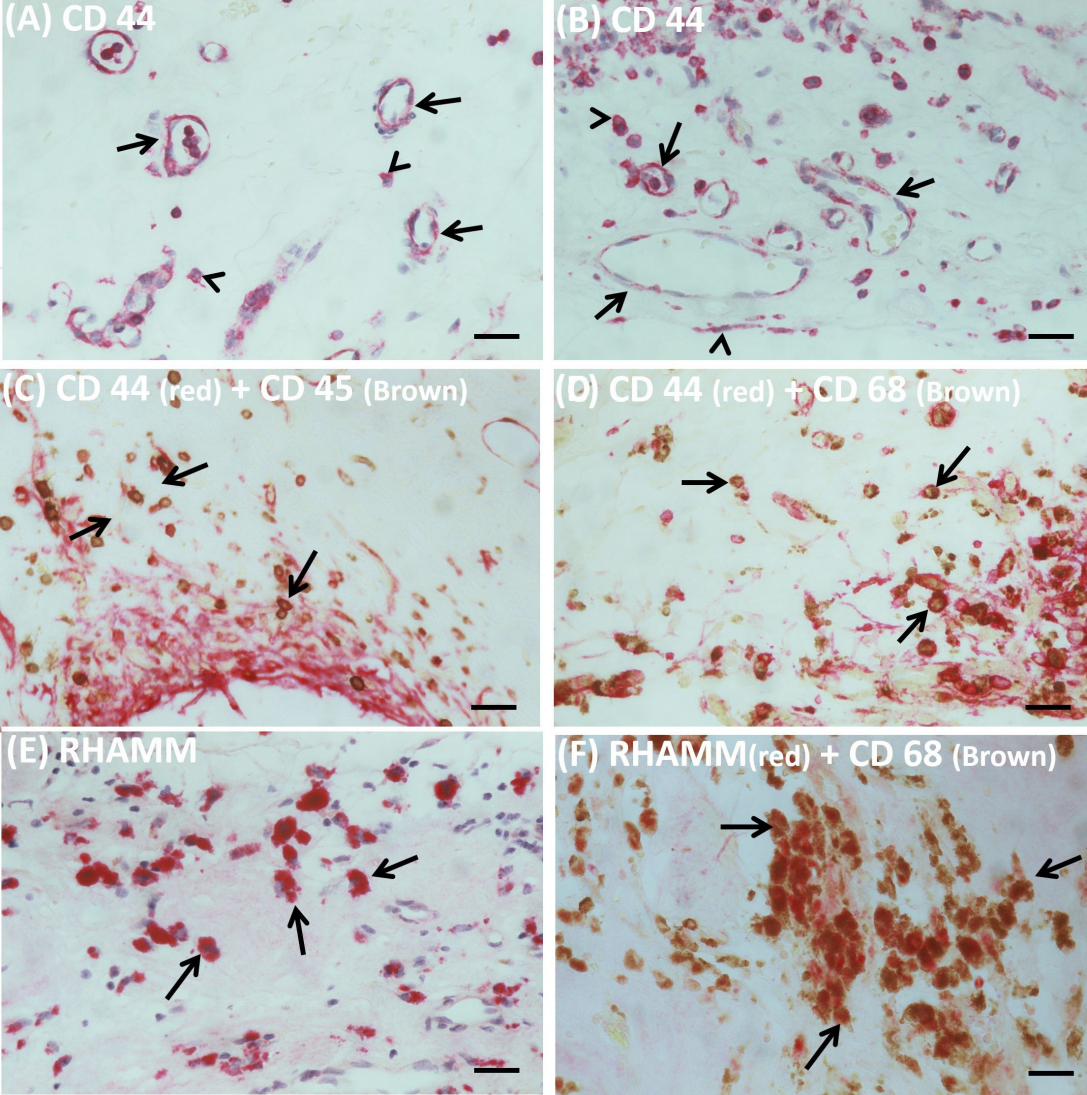
Immunoreactivity for CD44 was detected in vascular endothelial cells (arrows) and stromal cells (arrowheads) **(A, B)**. Stromal cells were leukocytes co-expressing CD45 (arrows) **(C)**, monocytes/macrophages co-expressing CD68 (arrows) **(D)** and spindle-shaped cells (arrowheads) **(B)**. Staining for receptor for hyaluronan-mediated motility (RHAMM) showed immunoreactivity in monocytes/macrophages (arrows) **(E, F)** (black scale bar 10 µm).

We studied by immunohistochemical analysis epiretinal fibrovascular membranes that were obtained from 14 patients with PDR during pars plana vitrectomy for the repair of tractional retinal detachment. We examined and localized the cell types producing HAS2, Hyal-2, CD44 and RHAMM *in vivo*. No staining was observed in the negative control slides in which the staining procedure was performed with omission of the primary antibody from the protocol (**Figure 1A**). Pathologic neovessels that were positive for the vascular endothelial cell marker CD31, reflecting angiogenesis activity, were detected in all membranes (**Figure 1B**). Monocytes/macrophages expressing CD68 (**Figure 1C**) and spindle shaped myofibroblasts expressing the marker α-SMA (**Figure 1D**) were detected in the stromal compartment. Immunoreactivity for HAS2 was detected in all membranes and was specifically localized in endothelial cells expressing CD31 and stromal cells (**Figure 1E**). Stromal cells were spindle-shaped myofibroblasts expressing α-SMA (**Figure 1F**), leukocytes co-expressing the leukocyte common antigen CD45 (**Figure 1G**) and monocytes macrophages co-expressing CD68 (**Figure 1H**). The stromal spindle-shaped cells expressing HAS2 (**Figure 1F**) are most probably myofibroblasts as their morphology is similar to the α-SMA expressing myofibroblasts as shown in **Figure 1D**. Immunoreactivity for Hyal-2 was observed in vascular endothelial cells lining neovessels (**Figure 1I**). Immunoreactivity for CD44 was detected in vascular endothelial cells (**Figures 2A, B**), intravascular leukocytes (**Figures 2A, B**), stromal leukocytes co-expressing CD45 (**Figure 2C**) and stromal monocytes/macrophages co-expressing CD68 (**Figure 2D**). RHAMM was expressed in stromal monocytes/macrophages (**Figure 2E**) co-expressing CD68 (**Figure 2F**).

**Figure 3:**
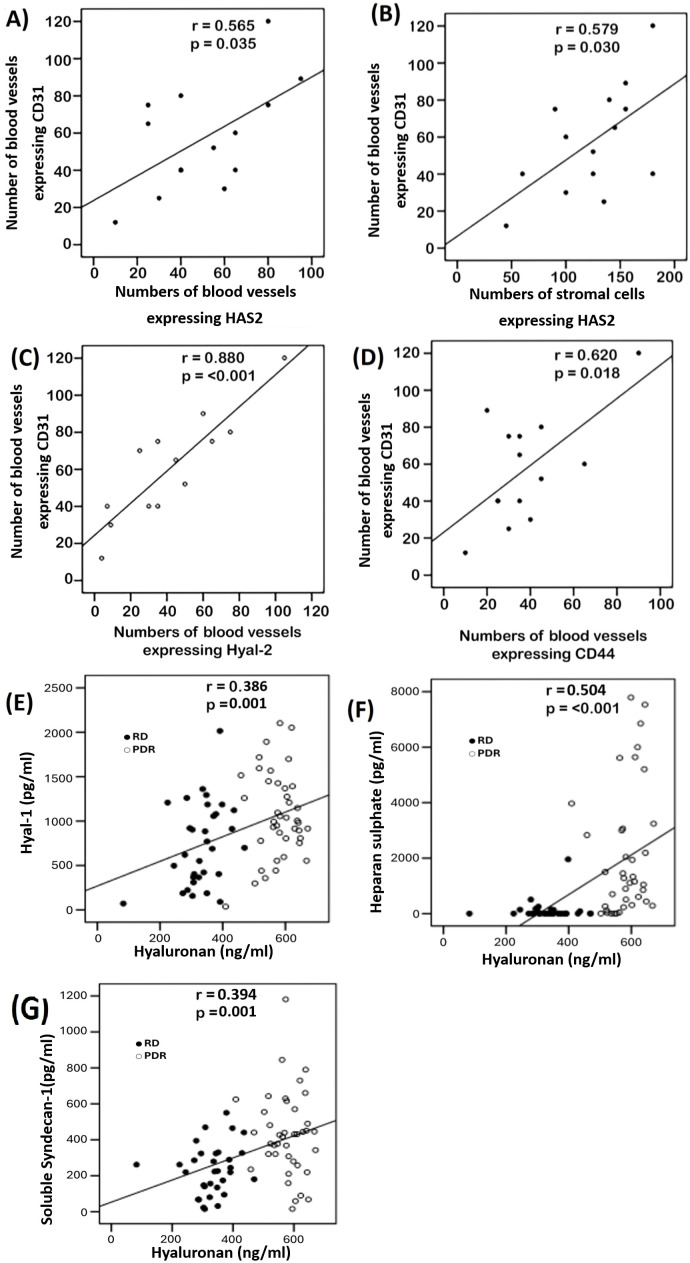
Immunohistochemical analysis of epiretinal fibrovascular membranes that were obtained from 14 patients with proliferative diabetic retinopathy during pars plana vitrectomy. Significant positive correlations between the numbers of blood vessels expressing CD31, reflecting angiogenic activity, and the numbers of blood vessels expressing hyaluronan synthase (HAS)2 **(A)**, the numbers of stromal cells expressing HAS2 **(B)**, the numbers of blood vessels expressing hyaluronidase (Hyal)-2 **(C)** and the numbers of blood vessels expressing CD44 **(D)** (Pearson’s correlation coefficient). Significant positive correlations between vitreous fluid levels of hyaluronan and the levels of hyaluronidase (Hyal)-1 **(E)**, heparan sulphate **(F)** and soluble syndecan-1 **(G)** (Pearson’s correlation coefficient). Samples from patients with proliferative diabetic retinopathy (PDR) are indicated by open circles, whereas samples from non-diabetic patients with rhegmatogenous retinal detachment (RD) are indicated by closed circles, illustrating sample clustering according to disease entity.

Significant positive correlations (Pearson’s correlation coefficient) were detected between numbers of pathologic neovessels expressing CD31, reflecting angiogenic activity, and the numbers of blood vessels (r=0.565; p=0.035) (**Figure 3A**) and stromal cells (r=0.579; p=0.03) expressing HAS2 (**Figure 3B**), numbers of blood vessels expressing Hyal-2 (r=0.88; p<0.001) (**Figure 3C**) and numbers of blood vessels expressing CD44 (r=0.62; p=0.018) (**Figure 3D**).

### 3.2 ELISA levels of Hyal-1, HA, syndecan-1 and heparan sulphate in vitreous samples from patients with PDR and nondiabetic control patients

We used ELISA to compare the levels of Hyal-1, HA, syndecan-1 and heparan sulphate in vitreous fluid samples from 40 patients with PDR to those of 32 nondiabetic clinical controls. Significant positive correlations (Pearson’s correlation coefficient) were found between the levels of HA and the levels of Hyal-1 (r=0.386; p=0.001) (**Figure 3E**), heparan sulphate (r=0.504; p<0.001) (**Figure 3F**) and syndecan-1 (r=0.394; p=0.001) (**Figure 3G**). As was also evident by the clustering of samples from different patient cohorts (**Figure 3**), the levels in vitreous samples from patients with PDR were significantly higher than the levels in nondiabetic control samples (p=0.004; p<0.001; p<0.001; p<0.001 for Hyal-1, HA, syndecan-1 and heparan sulphate, respectively) (**Table 1**).

**Table 1:** Comparisons of hyaluronidase-1 (Hyal-1), hyaluronan (HA), syndecan-1 and heparan sulphate levels in vitreous samples from patients with proliferative diabetic retinopathy (PDR) and nondiabetic patients with rhegmatogenous retinal detachment (RD).

Analyzed markers	PDR (n=40) mean± SD	RD (n=32) mean± SD	p-value (independent t-test)
Hyal-1 (pg/ml)	1067± 481	730.7± 463.5	0.004*
HA (ng/ml)	577.9± 58.3	330.8± 71.1	< 0.001*
Syndecan-1 (pg/ml)	427.8± 230.9	232.8± 138.1	< 0.001*
Heparan sulphate (pg/ml)	2023.5± 2300.3	107.5± 353.8	< 0.001*

*Statistically significant at 5% level of significance.

### 3.3 Western blot analysis of vitreous samples

**Figure 4:**
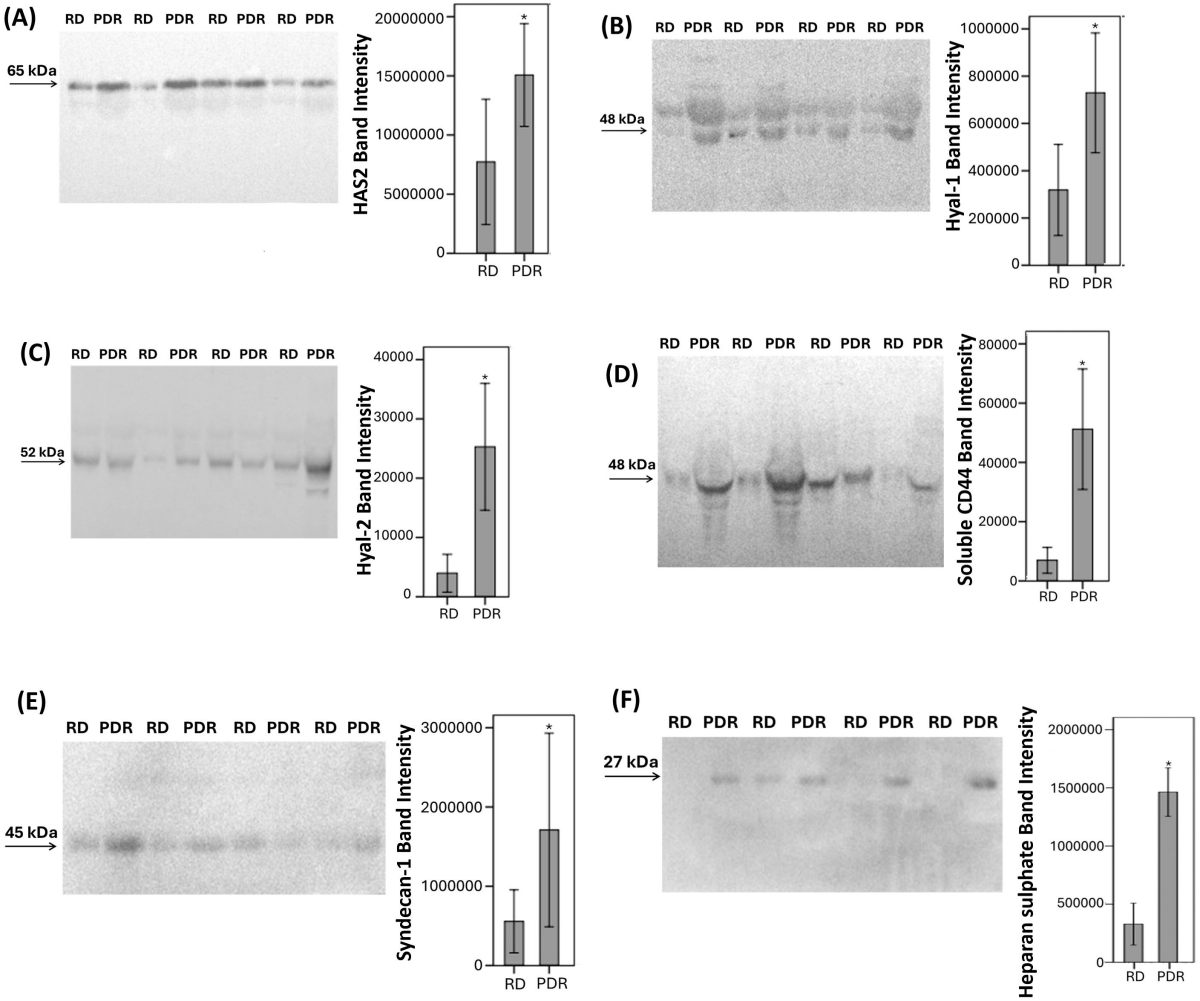
Determination of hyaluronan synthase (HAS)2 **(A)**, hyaluronidase (Hyal)-1 **(B)**, Hyal-2 **(C)**, soluble CD44 **(D)**, syndecan-1 **(E)** and heparan sulphate **(F)** levels in vitreous fluid samples. Equal volumes (15 µL) of vitreous fluid from patients with proliferative diabetic retinopathy (PDR; n=10) and from non-diabetic patients with rhegmatogenous retinal detachment (RD; n=10) were subjected to gel electrophoresis and the presence of HAS2, Hyal-1, Hyal-2, soluble CD44, syndecan-1, and heparan sulphate were detected by Western blot analysis. Representative sets of samples are shown. The intensity of the protein bands was determined in all samples and band intensities were compared between RD and PDR patients. Results are expressed as mean ± standard deviation or standard error of mean (*p < 0.05, independent t-test).

With the use of Western blot analysis of equal volumes of vitreous fluid we demonstrated the presence of the soluble (s) HAS2 (18) (**Figure 4A**), Hyal-1 (**Figure 4B**), Hyal-2 (**Figure 4C**), sCD44 (19) (**Figure 4D**), syndecan-1 (**Figure 4E**) and heparan sulphate (**Figure 4F**) in vitreous fluid samples from patients with PDR (n=10) and non-diabetic control patients (n=10). In contrast, RHAMM was not detected. Scanning analysis of immunoreactivities demonstrated significant increase in the levels of sHAS2, Hyal-1, Hyal-2, sCD44, syndecan-1 and heparan sulphate in vitreous of PDR patients in comparison with controls.

### 3.4 Effects of diabetes on retinal expression of Hyal-1, CD44, RHAMM and reactive oxygen species in streptozotocin-induced diabetic rats

**Figure 5:**
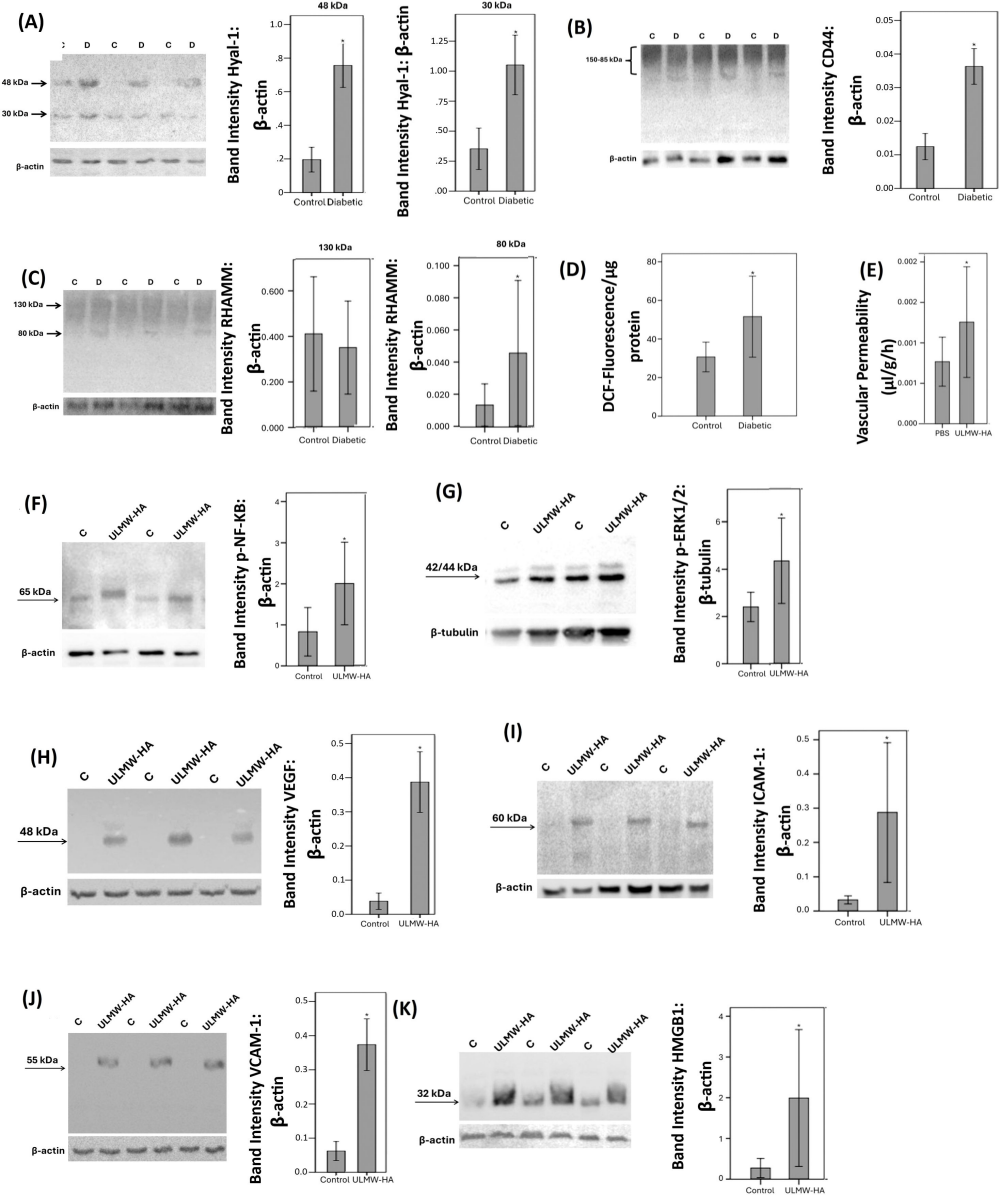
Expression levels of hyaluronidase (Hyal)-1 **(A)**, CD44 **(B)** and receptor for hyaluronan-mediated motility (RHAMM) **(C)** in the retinal lysates of non-diabetic control rats (C) (n=12) and diabetic rats (D) (n=12) were determined by Western blot analysis. After determination of the intensity of the protein bands, intensities were adjusted to those of β-actin in the samples. Oxidative stress was monitored with the use of 2’,7’-Dichlorofluorescein (DCF) fluorescence intensity analysis **(D)**. Results are expressed as mean ± standard deviation. Ultra-Low molecular weight hyaluronan (ULMW-HA) induces breakdown of blood-retinal barrier **(E)**. ULMW-HA was injected intravitreally at the dose of 50 ng in 5 µL in one eye and the same volume of phosphate-buffered saline (PBS) was injected in the contralateral eye of normal rats. The BRB was quantified with the fluorescein isothiocyanate-conjugated dextran technique. Results are expressed as mean ± standard deviation of 12 rats. *p < 0.05 compared to the values obtained from PBS-injected eyes. (independent t-test). Western blot analysis of retinas demonstrated that intravitreal injection of ULMW-HA induced significant upregulation of the expression of phospho-NF-κB **(F)**, phospho-ERK1/2 **(G)**, vascular endothelial growth factor (VEGF) **(H)**, intercellular adhesion molecule-1 (ICAM-1) **(I)**, vascular cell adhesion molecule-1 (VCAM-1) **(J)** and high-mobility group box-1 (HMGB1) **(K)**. Results are expressed as mean ± standard deviation or standard error of mean of 8–10 rats in each group (*p < 0.05; independent t-test).

Next, we studied the regulation of retinal expression of Hyal-1, CD44 and RHAMM in streptozotocin-induced diabetic rats. With the use of Western blot analysis of homogenized retinal tissue, we demonstrated that Hyal-1 was expressed as 2 protein bands at approximately 48 kDa and 30 kDa. Densitometric analysis showed increased Hyal-1 protein levels in the retina of rats after 4 weeks of STZ-induced diabetes (**Figure 5A**). CD44 immunoreactivities were expressed as multiple proteoforms at approximately 85–150 kDa. These corresponded to the CD44 standard isoform at around 85 kDa. The higher molecular weight proteins might represent CD44 variant isoforms as previously described (19, 20) and with the size heterogeneity of the attached glycosaminoglycans causing smearing on Western blot analysis. The CD44 variant isoforms were the most abundant isoform (**Figure 5B**). Scanning analysis of immunoreactivities demonstrated increased levels of CD44 protein levels in the retina of rats after 4 weeks of STZ-induced diabetes (**Figure 5B**). RHAMM was detected as 2 protein bands at approximately 130 kDa and 80 kDa (**Figure 5C**). Previous reports described the detection of different RHAMM isoforms due to alternative splicing (21). Densitometric analysis demonstrated significant upregulation of the 80 kDa protein band in the retina of diabetic rats (**Figure 5C**). **Figure 5D** shows the changes in DCF Fluorescence signal, which is an indication of reactive oxygen species (ROS) in the retina. We demonstrated a significant increase in DCF Fluorescence in the retina of rats after 4 weeks of STZ-induced diabetes compared with that of control animals.

### 3.5 Effect of intravitreal administration of ultra-low molecular weight/ULMW-HA on blood retinal barrier and on retinal expression of signaling pathways, proangiogenic factors and proinflammatory factors in normal rats

To investigate the *in vivo* effects of ULMW-HA, we used intravitreal injection in rats. Fluorescein isothiocyanate-conjugated dextran was used to assess the extent of the BRB breakdown. Intravitreal injection of ULMW-HA at the dose of 50 ng in 5 µl significantly increased retinal vascular permeability compared with PBS (vehicle)-injected eyes (**Figure 5E**). At the doses of 10 ng in 5 µl or 25 ng in 5 µl, ULMW-HA did not significantly increase retinal vascular permeability. Western blot analysis of homogenized retinal tissue was used to demonstrate that intravitreal injection of ULMW-HA induced significant upregulation of the protein levels of the signaling pathways phospho-NF-кB (**Figure 5F**) and phospho-ERK1/2 (**Figure 5G**), the proangiogenic factor VEGF (**Figure 5H**), the leukocyte adhesion molecules ICAM-1 (**Figure 5I**) and VCAM-1 (**Figure 5J**) and the proinflammatory alarmin HMGB1 (**Figure 5K**) compared to the values obtained from the contralateral eye that received vehicle (PBS) alone.

### 3.6 Effect of diabetic retinopathy-mimetic conditions on the expression of HAS2, Hyal-1, Hyal-2, CD44, RHAMM and HA in human retinal Müller glial cells and human retinal microvascular endothelial cells

**Figure 6:**
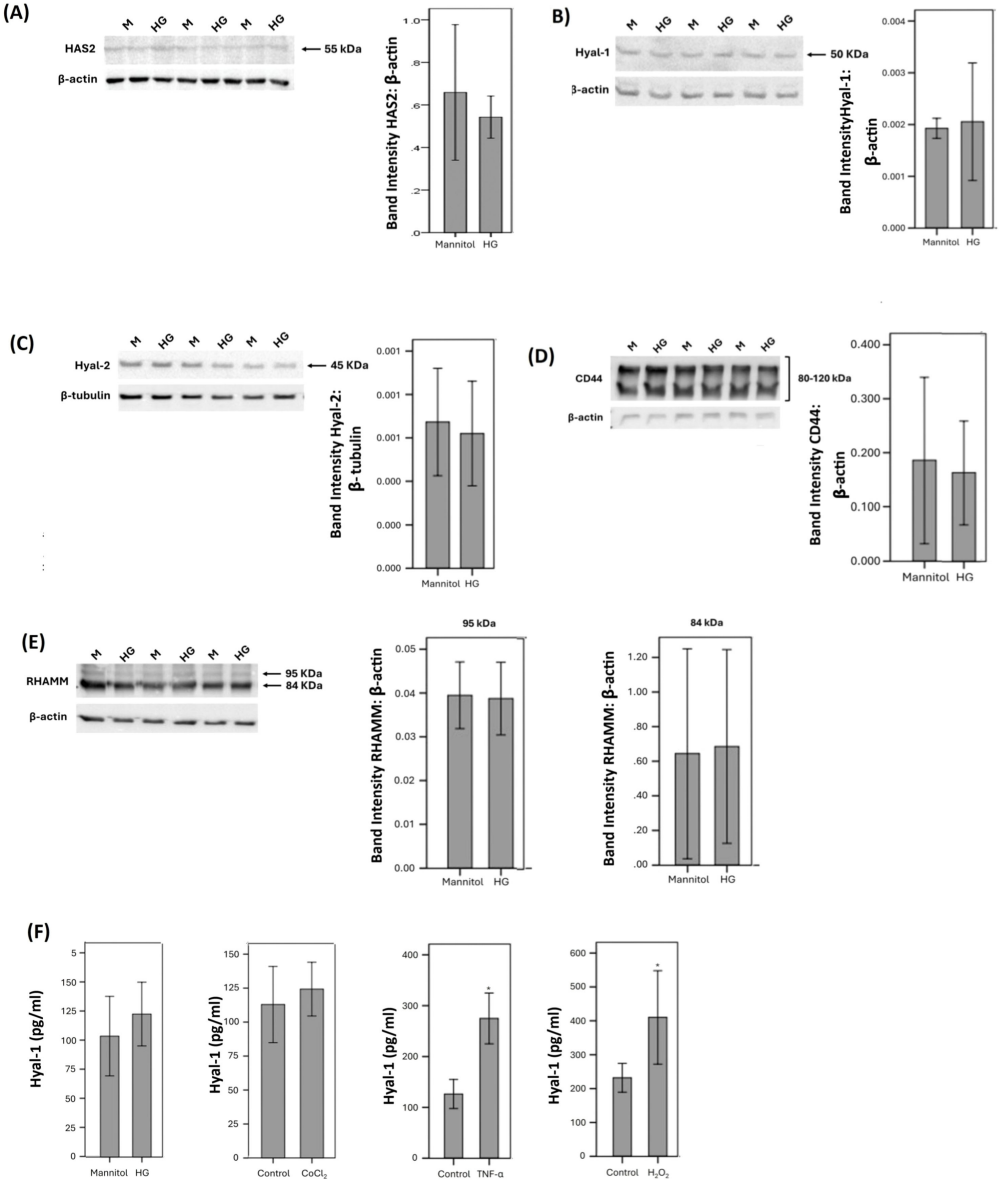
Human retinal Müller glial cells were left untreated or treated with high-glucose (HG) (25 mM) for 24 (h). For HG treatment, cultures treated with mannitol (25 mM) were used as a control. Protein expression of hyaluronan synthase (HAS)2 **(A)**, hyaluronidase (Hyal)-1 **(B)**, Hyal-2 **(C)**, CD44 **(D)** and receptor for hyaluronan-mediated motility (RHAMM) **(E)** in cell lysate was determined by Western blot analysis. Human retinal Müller glial cells were left untreated or treated with HG (25 mM), cobalt chloride (CoCl_2_) (300 µM), tumor necrosis factor-α (TNFα) (5 ng/mL) or hydrogen peroxide (H_2_O_2_) (10 mM) for 24 (h) Levels of hyaluronidase (Hyal)-1 were quantified in the culture media by ELISA. **(F)** Results are expressed as mean ± standard deviation or standard error of mean from three different experiments each performed in triplicate (*p < 0.05; independent t-test).

We performed *in vitro* experiments on Müller cells and HRMECs to demonstrate changes in molecules of the HA pathway by conditions mimicking diabetes. As analyzed with Western blot, Müller cells constitutively expressed HAS2 (**Figure 6A**), Hyal-1 (**Figure 6B**), Hyal-2 (**Figure 6C**), CD44 (**Figure 6D**), and RHAMM (**Figure 6E**). Treatment of Müller cells with the diabetic mimetic conditions high-glucose (HG), the hypoxia mimetic agent CoCl_2_, the proinflammatory cytokine TNF-α and the exogenous reactive oxygen species H_2_O_2_ did not affect the expression of HAS2, Hyal-1, Hyal-2, CD44 or RHAMM. **Figure 6** shows representative Western blot results for the expression of these proteins. With ELISA analysis, we revealed that treatment of Müller cells with TNF-α or H_2_O_2_ significantly increased the levels of Hyal-1 in the culture medium as compared to untreated control. However, HG or CoCl_2_ did not affect the levels of Hyal-1 in the cell culture supernatants (**Figure 6F**).

**Figure 7:**
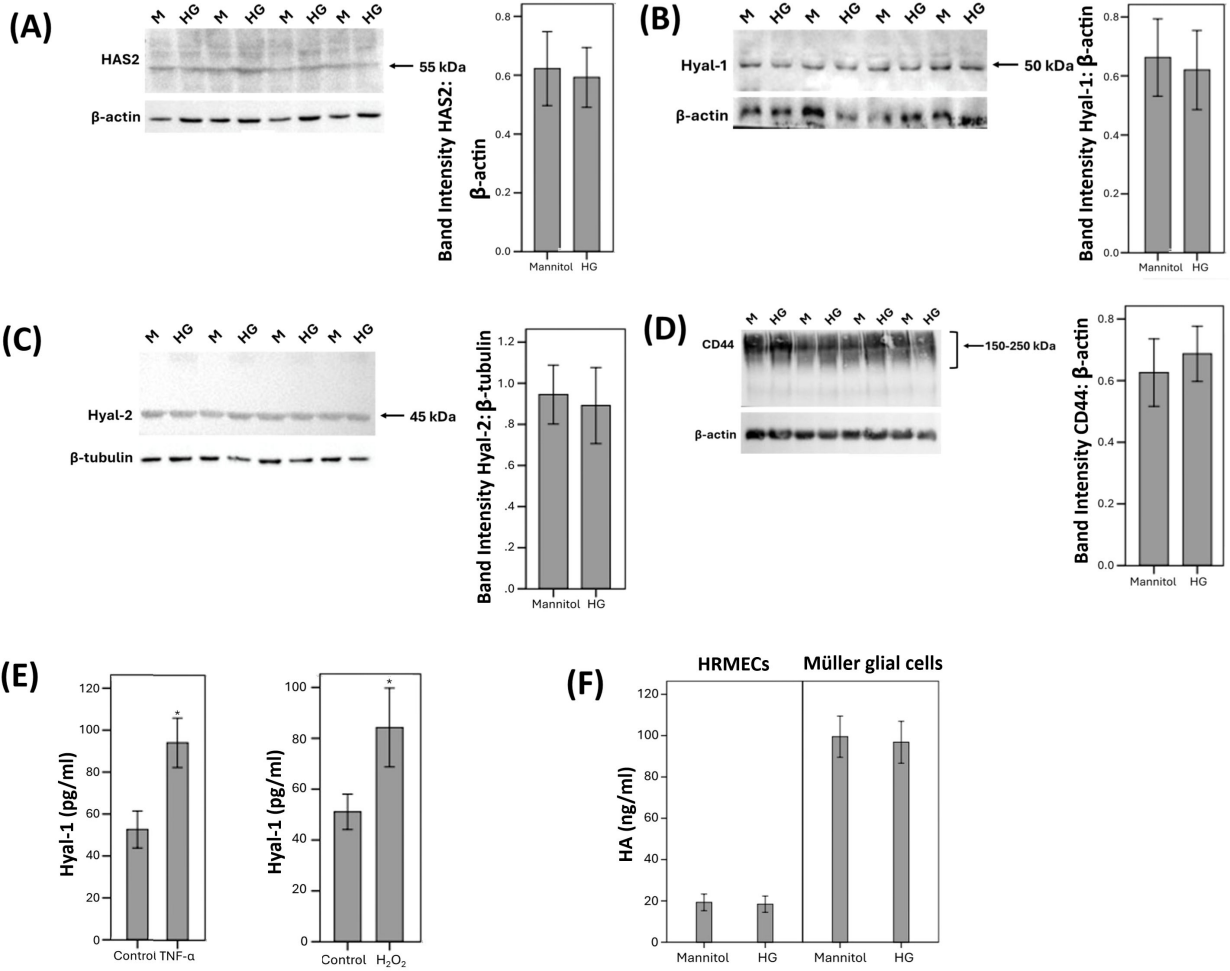
Human retinal microvascular endothelial cells (HRMECs) were left untreated or treated with high-glucose (HG) (25 mM) for 24h. For HG treatment, cultures treated with mannitol (25 mM) were used as a control. Protein expression of hyaluronan synthase (HAS)2 **(A)**, hyaluronidase (Hyal)-1 **(B)**, Hyal-2 **(C)** and CD44 **(D)** in cell lysate was determined by Western blot analysis. **(E)** HRMECs were left untreated or treated with tumor necrosis factor–α (TNF-α) (5ng/ml) or hydrogen peroxide (H_2_O_2_) (10mM) for 24h. Levels of Hyal-1 were quantified in the culture media by ELISA. **(F)** HRMECs and human retinal Müller glial cells were compared after treatment with mannitol (25 mM) or HG (25 mM) for 24h. Levels of hyaluronan (HA) were quantified in the culture media by ELISA. Results are expressed as mean ± standard deviation from three different experiments each performed in triplicate (*p < 0.05; independent t-test).

Western blot analysis was also used to demonstrate that HRMECs constitutively express HAS2 (**Figure 7A**), Hyal-1 (**Figure 7B**), Hyal-2 (**Figure 7C**), and CD44 (**Figure 7D**). However, HRMECs did not express RHAMM. These findings are in agreement with our immunohistochemical analysis findings in epiretinal fibrovascular membranes from patients with PDR (see above). Treatment of HRMECs with the studied diabetic mimetic conditions did not affect the expression of HAS2, Hyal-1, Hyal-2 or CD44. **Figure 7** shows representative Western blot results about the expression levels of these proteins. With ELISA analysis, we demonstrated that treatment of HRMECs with TNF-α or H_2_O_2_ significantly increased the levels of Hyal-1 in the culture medium as compared to untreated control (**Figure 7E**). However, HG or CoCl_2_ did not affect the levels of Hyal-1 in the culture medium (data not shown).

We next assessed the effect of the diabetic mimetic conditions on the detectable amounts of total HA levels in the culture medium of human retinal Müller glial cells and HRMECs. With the use of ELISA analysis, we demonstrated that Müller glial cells and HRMECs constitutively produce HA. However, treatment with the studied diabetic mimetic conditions did not affect HA levels in the culture medium (**Figure 7F**).

### 3.7 Effect of ultra-low molecular weight/ULMW-HA on human retinal Müller glial cells

**Figure 8:**
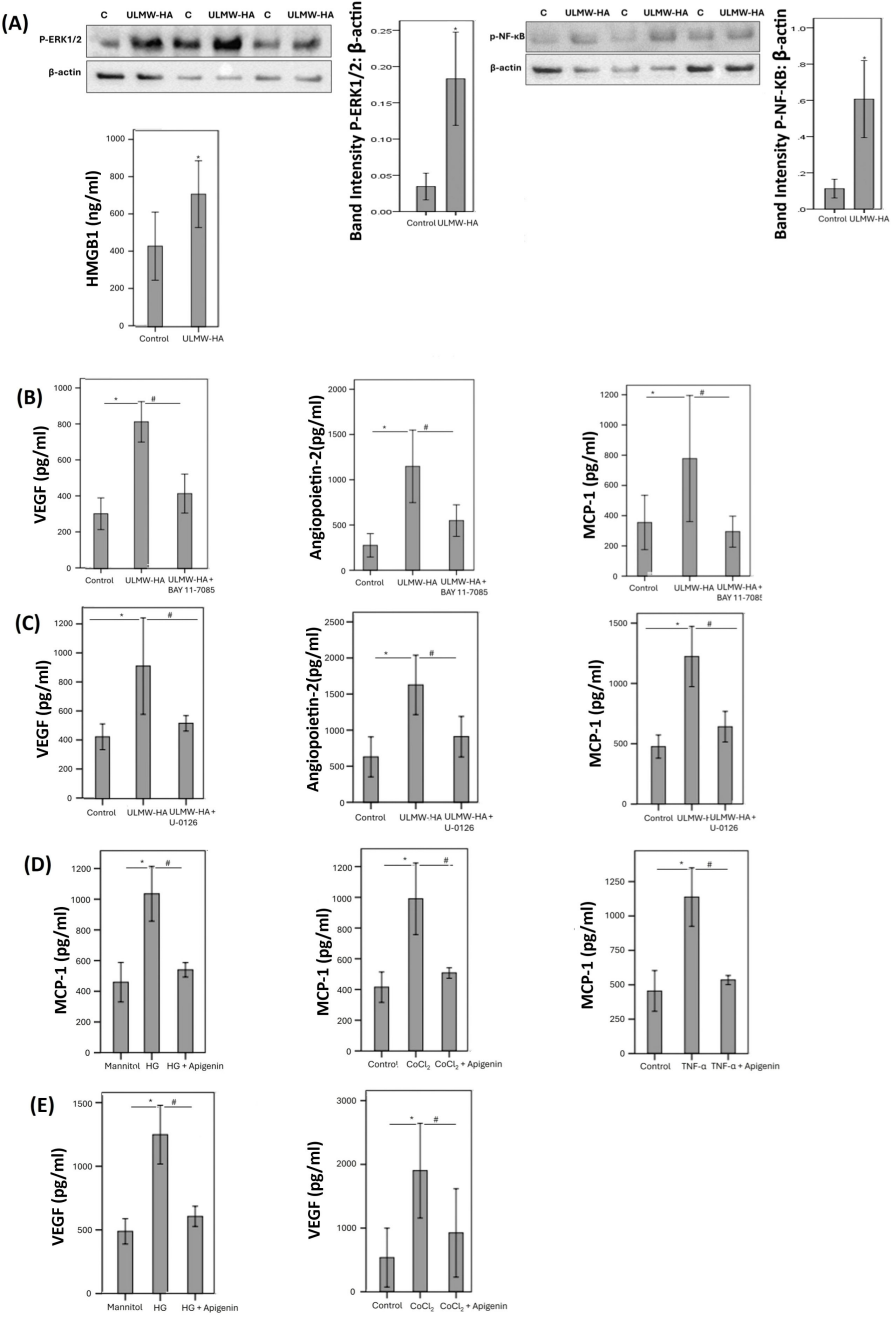
Human retinal Müller glial cells were left untreated or treated with ultra-low molecular weight hyaluronan (ULMW-HA) (50 µg/mL) for 24 (h) **(A)** Protein expression of phospho-ERK1/2 and phospho-NFκB in cell lysates was determined by Western blot analysis. Levels of high mobility group box-1 (HMGB1) were quantified in the culture media by ELISA. Results are expressed as mean ± standard deviation from three different experiments each performed in triplicate (*p < 0.05; independent t-test). **(B)** Human retinal Müller glial cells were left untreated or treated with ULMW-HA, ULMW-HA plus BAY11-7085 (5 µM) or **(C)** ULMW-HA plus U-0126 (5 µM). Levels of vascular endothelial growth factor (VEGF), angiopoietin and monocyte chemotactic protein-1 (MCP-1/CCL2) were quantified in the culture media by ELISA. Results are expressed as mean ± standard deviation or standard error of mean from three different experiments each performed in triplicate. One-way ANOVA and independent t-test were used for comparisons between three groups and two groups, respectively. *p < 0.05 compared with values obtained from untreated cells; #p < 0.05 compared with ULMW-HA plus BAY11–7085 or U-0126 treated cells. **(D, E)** Human retinal Müller glial cells were left untreated or treated with high glucose (HG) (25 mM), cobalt chloride (CoCl_2_) (300 µM) or tumor necrosis factor-α (TNF-α) (5 ng/mL) with or without apigenin (10 µg/mL) for 24 (h) For HG treatment, cultures containing 25 mM mannitol were used as a control. Levels of monocyte chemotactic protein-1 (MCP-1/CCL2) **(D)** and vascular endothelial growth factor (VEGF) **(E)** were quantified in the culture media by ELISA. The results are expressed as mean ± standard deviation from three different experiments each performed in triplicate. One-way ANOVA and independent t-test were used for comparisons between three and two groups, respectively. *p < 0.05 compared with values obtained from control cells. #p < 0.05 compared with values obtained from stimulated cells.

Western blot analysis revealed that treatment of cultured Müller cells with exogenous ULMW-HA induced significant upregulation of the protein levels of the signaling pathways phospho-ERK1/2 and phospho-NF-кB as compared to untreated control. The changes in these signaling events were corroborated with a functional read-out, as ELISA analysis revealed that ULMW-HA significantly increased the levels of the potent proinflammatory alarmin HMGB1 in the culture medium (**Figure 8A**). In addition, ELISA analysis revealed that treatment of cultured Müller cells with exogenous ULMW-HA significantly increased the levels of the proangiogenic factors VEGF and angiopoietin 2 and the inflammatory chemokine MCP-1/CCL2 in the culture medium as compared to untreated control (**Figure 8B**). However, treatment with ULMW-HA did not affect the levels of MMP-9 in the culture medium as compared to untreated control. Co-treatment with ULMW-HA plus the NF-кB inhibitor BAY11–7085 significantly attenuated ULMW-HA-induced upregulation of VEGF, angiopoietin and MCP-1/CCL2 in Müller cells (**Figure 8B**). In addition, inhibition of the ERK1/2 signaling pathway by U-0126 significantly reduced VEGF, angiopoietin 2 and MCP-1/CCL2 induced by ULMW-HA (**Figure 8C**).

### 3.8 Effect of the hyaluronidase inhibitor apigenin on diabetes-mimetic upregulation of proangiogenic and proinflammatory factors in human retinal Müller glial cells

ELISA analysis demonstrated that treatment of Müller cells with HG, CoCl_2_, and TNF-α induced significant upregulation of the inflammatory chemokine MCP-1/CCL2 in the culture medium as compared to untreated control. Pretreatment of Müller cells with apigenin significantly reduced levels of MCP-1/CCL2 induced by HG, CoCl_2_, and TNF-α (**Figure 8D**). Treatment of Müller cells with HG and CoCl_2_ induced significant upregulation of the proangiogenic factor VEGF in the culture medium as compared to untreated control. Pretreatment with apigenin significantly reduced the levels of VEGF induced by HG and CoCl_2_ (**Figure 8E**).

### 3.9 Effect of the hyaluronidase inhibitor apigenin on TNF-α-induced THP-1 monocyte adhesion to human retinal microvascular endothelial cells

**Figure 9:**
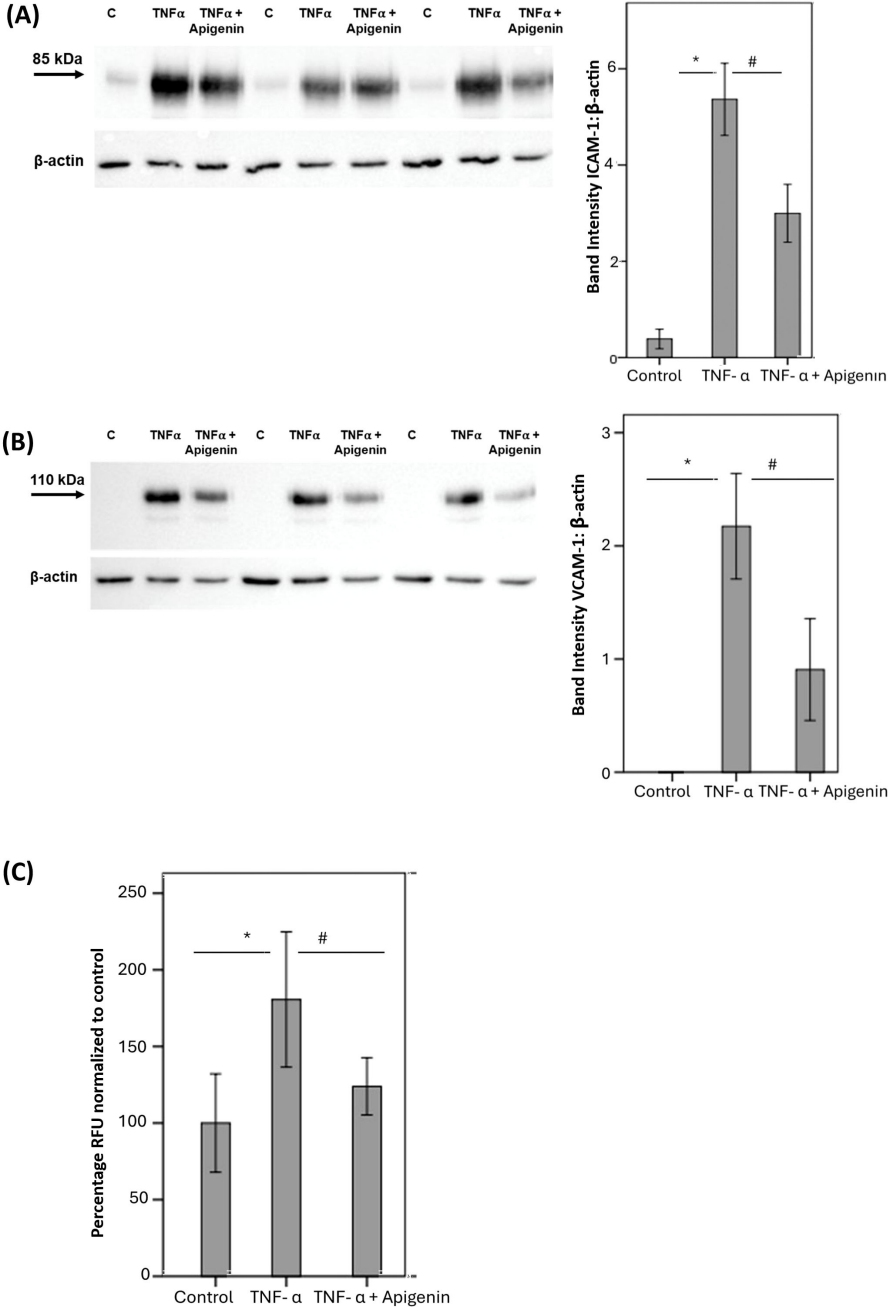
Human retinal microvascular endothelial cells (HRMECs) were left untreated or stimulated with tumor necrosis factor-α (TNF-α) (5 ng/mL) for 24 h with or without apigenin (10 µg/mL). Protein expression of intercellular adhesion molecule-1 (ICAM-1) **(A)** and vascular cell adhesion molecule-1 (VCAM-1) **(B)** was determined by Western blot analysis. Adhesion of fluorescently labeled THP-1 monocytic cells to HRMECs monolayer was quantified **(C)**. Results are expressed as mean ± standard deviation or standard error of mean from three different experiments each performed in triplicate. One-way ANOVA and independent t-test were used for comparisons between three groups and two groups, respectively. *p < 0.05 compared with values obtained from untreated cells. #p < 0.05 compared with values obtained from cells treated with TNF-α (RFU = relative fluorescence unit).

Pretreatment of HRMECs with apigenin significantly reduced TNF-α induced upregulation of the leukocyte adhesion molecules ICAM-1 (**Figure 9A**) and VCAM-1 (**Figure 9B**). Furthermore, pretreatment of HRMECs with apigenin significantly decreased TNF-α induced adherence of THP-1 monocytes to HRMECs (**Figure 9C**).

### 3.10 Effect of the hyaluronidase inhibitor apigenin on the shedding of the endothelial glycocalyx component soluble syndecan-1 induced by diabetic retinopathy-associated mechanisms in human retinal microvascular endothelial cells

**Figure 10:**
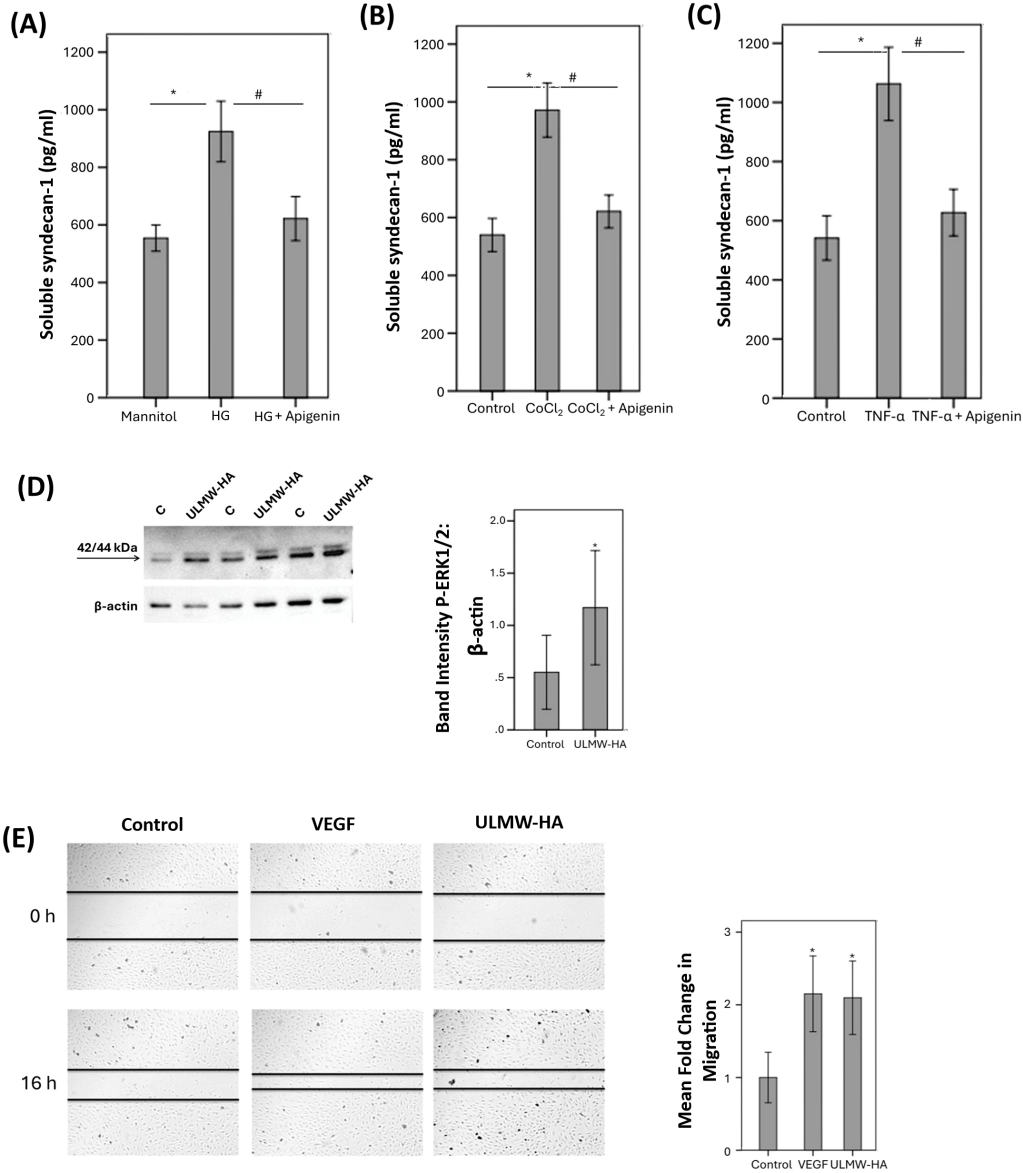
Human retinal microvascular endothelial cells (HRMECs) were left untreated or treated with high glucose (HG) (25 mM) **(A)**, cobalt chloride (CoCl_2_) (300 µM) **(B)** or tumor necrosis factor-α (TNF-α) (5 ng/mL) **(C)** with or without apigenin (10 µg/mL). For HG treatment, cultures treated with mannitol (25 mM) were used as a control. Levels of soluble syndecan-1 were quantified in the culture media by ELISA. Results are expressed as mean ± standard deviation from three different experiments each performed in triplicate. One-way ANOVA and independent t-test were used for comparisons between three and two groups, respectively. *p < 0.05 compared with values obtained from control cells. #p < 0.05 compared with values obtained from cells treated with HG, CoCl_2_ or TNF-α. HRMECs were left untreated or were stimulated with ultra-low molecular weight – hyaluronan (ULMW-HA) (50 µg/mL) for 24 (h). Protein expression of phospho-ERK1/2 in the cell lysates was determined by Western blot analysis **(D)**. Results are expressed as mean ± standard deviation from three different experiments each performed in triplicate (*p < 0.05; independent t-test). A scratch was performed in confluent monolayers of overnight starved HRMECs with a micropipette tip subsequently, the cultures were left untreated or treated either with VEGF (10 ng/mL) or with ULMW-HA (100 µg/mL) for 16 (h) Cells were visualized using an inverted microscope. Two independent experiments were performed. Each experiment was done in duplicate, and 2–3 independent field images were taken for the migration analysis which was done by using Image J software. In the Figure, one representative image is illustrated, and the bar graphs show the analysis of all the images from each group represented as fold-change in migration versus control **(E)**. Results are expressed as mean ± standard deviation. One-way ANOVA and independent t-test were used for comparisons between three and two groups, respectively. *p < 0.05 compared with values obtained from control cells.

With the use of ELISA analysis, we showed that treatment of HRMECs with the studied diabetes-mimetic conditions induced significant upregulation of soluble syndecan-1 in the culture medium as compared to untreated control. Pretreatment with the hyaluronidase inhibitor apigenin significantly attenuated the levels of soluble syndecan-1 induced by HG (**Figure 10A**), CoCl_2_ (**Figure 10B**) and TNF-α (**Figure 10C**).

### 3.11 Effect of ultra-low molecular weight/ULMW-HA on human retinal microvascular endothelial cells

Western blot analysis demonstrated that treatment of HRMECs with ULMW-HA at a concentration of 50 µg/mL induced significant upregulation of phospho-ERK1/2 (**Figure 10D**). Migration of endothelial cells is a key step in the angiogenesis cascade. We tested ULMW-HA for its ability to induce migration of HRMECs. ULMW-HA at a concentration of 100 µg/mL significantly induced migration of HRMECs and was as potent as 10 ng/mL of VEGF (**Figure 10E**).

## 4 Discussion

In the present study, we showed for the first time that the levels of the enzyme HAS2 and its reaction product HA were significantly upregulated in the vitreous fluid from the patients with PDR. Immunohistochemical analysis demonstrated HAS2 protein expression by endothelial cells lining pathologic new blood vessels, myofibroblasts and monocytes/macrophages in the epiretinal fibrovascular membranes from patients with PDR. In addition, we demonstrated a significant positive correlation between the numbers of blood vessels expressing CD31, reflecting the angiogenic activity of PDR epiretinal fibrovascular membranes, and the expression of HAS2. We also demonstrated that cultured HRMECs and retinal Müller glial cells constitutively express HAS2 and produce HA. Similarly, previous studies reported that overexpression of HAS2 by tumors is associated with increased tumor HA production and enhanced tumor growth and angiogenesis (11–13, 22).

We also demonstrated upregulation of the HA-degradative enzymes Hyal-1 and Hyal-2 in the ocular microenvironment of patients with PDR. Using immunohistochemical analysis, we demonstrated that Hyal-2 was specifically localized in endothelial cells lining pathologic new blood vessels in epiretinal fibrovascular membranes from patients with PDR. We also demonstrated a significant positive correlation between the angiogenic activity in PDR fibrovascular epiretinal membranes and the level of Hyal-2 expression. Consistent with our results in clinical samples, we showed that Hyal-1 was also significantly upregulated in the retina of streptozotocin-induced diabetic rats. To corroborate the findings at the cellular level, we demonstrated that human retinal Müller glial cells and HRMECs upregulated their expression of Hyal-1 under diabetes-associated mechanisms, including the presence of the inflammatory cytokine TNF-α and H_2_O_2-_induced oxidative stress. Elevated levels of hyaluronidases and reactive oxygen species (ROS) (23, 24) within the ocular microenvironment of patients with PDR predict the accumulation of small HA fragments with low molecular weight as HA fragmentation is conducted enzymatically by hyaluronidases or nonenzymatically in the presence of ROS (13, 14, 22). The breakdown of HMW-HA causes the formation of smaller processed fragments that stimulate the expression of pro-inflammatory cytokines and growth factors. In addition, LMW-HA has angiogenic properties, whereas, endogenous HMW-HA has been shown to be anti-inflammatory and anti-angiogenic (12). Previous *in vivo* studies demonstrated that expression of hyaluronidases by tumor cells induced angiogenesis (25) and that enhanced expression of hyaluronidases in tumor cells induces tumor cell proliferation, migration, invasion, and angiogenesis (11). *In vitro* studies demonstrated that hyaluronidase stimulated the proliferation and migration of endothelial cells as well as tube-like structures in endothelial cells (26, 27), key steps in the angiogenesis cascade. It is possible that hyaluronidase induces endothelial cell tube formation through the formation of LMW-HA fragments with angiogenic activity (27).

Endothelial glycocalyx (EG) damage and degradation generate HA, heparan sulphate and syndecan-1, which are used as biomarkers of EG shedding and endothelial cell injury (15, 17). In the present study, we demonstrated elevated levels of HA and heparan sulphate glycosaminoglycans and syndecan-1 protein in the vitreous fluid from patients with PDR. Our observations thereby are in line with retinal endothelial cell injury and dysfunction in patients with PDR. In addition, positive correlations were demonstrated between vitreous fluid levels of HA and the levels of Hyal-1, syndecan-1 and heparan sulphate. We also demonstrated that treatment of cultured HRMECs with the diabetic retinopathy-associated mechanisms HG, CoCl_2_ and TNF-α induced significant upregulation of syndecan-1 in the culture medium. Previous studies demonstrated that hyperglycemia reduces thickness of EG and stimulates shedding of EG leading to increased vascular permeability (28–31). A previous study demonstrated that Hyal-1 contributes to EG dysfunction induced by diabetes. Hyal-1-deficient streptozotocin-induced diabetic mice display a thicker EG and are protected from endothelial dysfunction when compared with Hyal-1-competent mice (32). These findings suggest that Hyal-1 is a major actor in early diabetic vascular complications and that EG damage may play a central role in the pathogenesis of diabetic retinopathy.

A point of discussion relates to the molecular weights of the substrate and reaction products of hyaluronidases acting on HMW-HA. The substrate HMW-HA contains a mixture of molecules in the size-range of 1000 to 6000 kDa. When this polysaccharide biomaterial is degraded by hyaluronidases, at first a mixture of molecules is generated known in the literature as LMW-HA, having sizes less than 250 kDa and being stainable as a smear of polysaccharides upon gel electrophoresis. Further enzymatic degradation leads to mixtures of HA with gradually smaller molecular weights. Inherent to this insight is the fact that, by the natural process of catalysis of HA, one is always dealing with mixtures of HA of varying lengths. For the study of HA as agonist in biological processes it is therefore important to take the molecular weights of the used HA into consideration if one aims at reproducibility of experiments. For this reason, we did not use in-house generated preparations of LMW-HA, because these are often ill-defined and without biochemical characterization of molecular sizes and presence of contaminants, including endotoxins. Instead, we used a commercial preparation known as ultra-low molecular weight HA/ULMW-HA, that is biochemically characterized and available for comparative studies. This ULMW-HA preparation generated reproducible and clear effects *in vivo* and *in vitro*.

Retinal endothelial dysfunction and breakdown of blood-retinal barrier (BRB) are consistent findings underlying the pathophysiology of diabetic retinopathy (1–4). We demonstrated that intravitreal administration of ULMW-HA in normal rats significantly increased retinal vascular permeability. Additionally, intravitreal ULMW-HA induced signaling by activation of the proinflammatory transcription factor NF-кB and the extracellular signal-regulated kinase ERK1/2 and the synthesis of the potent proangiogenic factor VEGF, the proinflammatory alarmin HMGB1 and the leukocyte adhesion molecules ICAM-1 and VCAM-1. Our findings suggest that ULMW-HA-induced BRB breakdown might be related to upregulation of VEGF as VEGF is a major contributor to BRB breakdown in diabetic retinopathy (33–35) and is considered as the most potent pro-angiogenic factor in PDR (36, 37). To corroborate the findings at the cellular level, we demonstrated that treatment of Müller cells with ULMW-HA induced upregulation of phospho-ERK1/2 and activation of NF-кB and upregulated the expression of the proangiogenic factors VEGF and angiopoietin-2, the inflammatory chemokine MCP-1/CCL2 and the proinflammatory alarmin HMGB1. Müller cells are a major source of angiogenic factors secretion and therefore contribute to the development of pathological retinal angiogenesis (38). Our findings suggest that ULMW-HA stimulates Müller glial cells to secrete various pro-angiogenic and pro-inflammatory factors to promote the progression of PDR. Mechanistic studies demonstrated that ULMW-HA-induced upregulation of VEGF, angiopoietin-2 and MCP-1/CCL2 is mediated through ERK1/2 and NF-кB activation as the ERK1/2 inhibitor U-0126 and the NF-кB inhibitor BAY11–7085 attenuated ULMW-HA-induced upregulation of these pro-angiogenic and pro-inflammatory factors. Our findings are line with those of previous reports demonstrating that LMW-HA elicits pro-angiogenic and pro-inflammatory responses by activating ERK1/2 and NF-кB signaling pathways (21, 22, 39, 40). Additionally, previous reports demonstrated that LMW-HA promotes monocyte recruitment and differentiation of M1 subtype into M2 subtype enhancing M2 monocyte accumulation (41). In previous studies, we demonstrated that most of the CD68^+^ monocytes/macrophages in the ocular microenvironment of patients with PDR exhibit an M2 phenotype and that there is a significant positive correlation between the numbers of monocytes/macrophages and the degree of angiogenic activity (6, 42, 43).

LMW-HA is also involved in regulating vascular biology. *In vitro* studies demonstrated that LMW-HA induced vascular endothelial cell proliferation, migration and tubule formation, key steps in the angiogenesis process, as well as in various *in vivo* models of angiogenesis (22). Similarly, we demonstrated that ULMW-HA induced migration of HRMECs. The biological activities of LMW-HA are mediated through the cell surface receptors CD44 and receptor for HA-mediated motility (RHAMM, CD168). LMW-HA interaction with CD44 and RHAMM activates several signaling pathways that are involved in LMW-HA-mediated inflammation and angiogenesis (16, 40). We here demonstrated upregulation of CD44 and RHAMM in the retina of streptozotocin-induced diabetic rats and that Müller cells constitutively express CD44 and RHAMM. In epiretinal fibrovascular membranes from patients with PDR, CD44 and RHAMM were localized in monocytes/macrophages. In contrast, endothelial cells lining pathologic new blood vessels expressed CD44, but not RHAMM. Consistent with our results in clinical samples, we demonstrated that cultured HRMECs expressed CD44, but not RHAMM. These findings suggest preferential roles of the CD44 pathway in modulating PDR angiogenesis. Several studies reported the critical roles of CD44 in promoting pathological angiogenesis through its regulation of endothelial cell proliferation, migration, and adhesion to extracellular matrix components. Additionally, CD44 can enhance the adhesion of leukocytes to endothelial cells facilitating leukocyte infiltration (24). Thus, targeting CD44 or its ligands could become a therapeutic approach in patients with PDR.

Apigenin, a naturally occurring flavonoid in fruit and vegetables, has been described as a hyaluronidase inhibitor (44). In addition, apigenin was demonstrated to have significant anti-inflammatory activity (26, 45). In the present study, we demonstrated that treatment of cultured Müller cells with apigenin significantly downregulated the levels of the produced angiogenic factor VEGF and the inflammatory chemokine MCP-1/CCL2 induced by the studied diabetes-mimetic conditions. In addition, treatment of cultured HRMECs with apigenin significantly reduced TNF-α-induced upregulation of the leukocyte adhesion molecules ICAM-1 and VCAM-1 and significantly decreased binding of human monocytic THP-1 cells to HRMECs. Enhanced adhesion of circulating leukocytes to the retinal microvascular endothelium is a crucial element for the development of diabetes-induced retinal endothelial cell damage and breakdown of BRB (4). We also demonstrated that treatment of cultured HRMECs with apigenin significantly reduced EG damage and shedding of the glycocalyx component syndecan-1 into the culture medium after induction by diabetes-mimetic conditions.

In conclusion, we demonstrated abnormal HA metabolism in the intraocular microenvironment of patients with PDR. The dissection of protective HMW-HA and detrimental ULMW-HA needs more scrutiny, in particular because mixtures of HA are clinically used. Treatment approaches that target HA-mediated signaling events that promote diabetes-induced retinal endothelial dysfunction as well as inflammation and angiogenesis, many of which are mediated by ULMW-HA, might improve the efficacy of existing approaches to treat diabetic retinopathy. In addition, our findings stimulate studies of HA metabolism and signaling events in inflammatory pathologies beyond eye diseases with the use of approaches outlined here.

## Data Availability

The data analyzed in this study is subject to the following licenses/restrictions: The data presented in this study are available on request from the corresponding author. Requests to access these datasets should be directed to Ahmed M. Abu El-Asrar, abuelasrar@yahoo.com.
